# Giant Pilomatrixoma: Report of Two Cases and Review of Diagnostic and Therapeutic Approach

**DOI:** 10.7759/cureus.114693

**Published:** 2026-08-17

**Authors:** Bryan Salvador Sol Tlapalamatl, Andrea Zaragoza Navarro, María Fernanda Limon Limon, Teresa Jannete Ortega Valls, Amara Hazel Solorio Rivera, Priscilla Rojas Romero Hicks, José Alfredo Soto-Ortiz

**Affiliations:** 1 Dermatology, Instituto Dermatológico de Jalisco "Dr. José Barba Rubio", Zapopan, MEX; 2 General Medicine, Red Medica de la Universidad de Guanajuato, Guanajuato, MEX

**Keywords:** benign adnexal neoplasm, calcifying epithelioma, giant pilomatrixoma, hair follicle matrix tumor, pilomatrixoma

## Abstract

Pilomatrixoma is a benign adnexal neoplasm derived from hair matrix cells. Giant pilomatrixoma (GP) is an uncommon clinical variant that may pose a diagnostic challenge because of its resemblance to soft tissue neoplasms. We report two cases of GP with distinct clinical presentations. The first case involved a 22-year-old woman presenting with a right suprascapular mass measuring 14 × 8 × 5 cm, with slow progressive enlargement over three years. Magnetic resonance imaging revealed a well-circumscribed lesion with calcification foci. Complete surgical excision was performed, and histopathological examination demonstrated extensive calcium deposits, abundant ghost cells, and scarce basaloid cells, confirming an advanced-stage GP. The second case corresponded to a 54-year-old man with a four-year history of a left retroauricular mass measuring 6.5 × 4.5 × 3 cm. Surgical excision and histopathological analysis confirmed pilomatrixoma. Both patients had favorable postoperative outcomes without recurrence during follow-up. These cases highlight the variable clinical presentation of GP and the diagnostic challenge posed by its large size and atypical presentation. They emphasize the importance of clinicoradiologic and histopathologic correlation for establishing an accurate diagnosis and guiding appropriate surgical management.

## Introduction

Pilomatrixoma is a benign adnexal tumor first described in 1880 by Malherbe and Chenantais as calcifying sebaceous epithelioma, since it was initially believed to originate from sebaceous glands [1]. Subsequently, in 1961, Forbis and Helwig demonstrated its origin from hair matrix cells, establishing the basis for its current nomenclature [2].

Its prevalence remains unknown and it is estimated to account for less than 1% of all benign tumors; however, it is considered one of the most common follicular neoplasms [3,4]. It occurs more frequently in women during the first two decades of life, with a second peak occurring between the fifth and sixth decades [4-6].

Regarding its etiopathogenesis, alterations in the Wnt signaling pathway have been proposed, involving genes such as CTNNB1 and BCL2. In this context, somatic mutations, particularly in exon 3 of CTNNB1, have been identified in more than 75% of cases, leading to nuclear accumulation of β-catenin and promoting proliferation of matricial cells [3,4,6].

Clinically, pilomatrixoma presents as a solitary dermal or subcutaneous neoplasm with a firm to rock-hard consistency, smooth surface, and coloration similar to the surrounding skin. It most commonly involves the head, neck, and upper extremities, with a usual size ranging from 0.5 to 3 cm in diameter [6,7].

Multiple clinical variants have been described. Despite their shared histogenesis, pilomatrixomas may exhibit considerable clinicopathological variability regarding size, anatomical distribution, age at presentation, and degree of tumor maturation. Long-standing lesions frequently demonstrate advanced histopathological changes, including extensive calcification, predominance of ghost cells, and progressive reduction of the basaloid component, reflecting late stages of tumor evolution. In this spectrum, giant pilomatrixoma (GP), first reported in 1974 by Krausen et al., is defined as a lesion exceeding 4-5 cm in diameter [8-10]. This variant represents an uncommon form compared with conventional presentations previously described. It is usually associated with prolonged evolution, progressive growth, and higher degree of tumor calcification [9-12]. In the literature, reported cases of GP show a predilection for the trunk, particularly the posterior thoracic region, whereas the non-giant form predominantly affects the cephalic region [10-13].

From a clinical standpoint, its large size, stony consistency, and occasional presence of irregularities or vascular changes may mimic malignant soft tissue neoplasms, frequently leading to initial diagnostic errors [9,11,14]. Furthermore, GPs may exhibit histopathological patterns characterized by predominance of ghost cells, extensive calcification, and even ossification, corresponding to late stages of tumor evolution [12-15]. The differential diagnosis includes benign lesions such as epidermoid cysts and calcified lipomas, as well as malignant neoplasms such as dermatofibrosarcoma protuberans and pilomatrical carcinoma, among other calcified soft-tissue tumors [9,14].

Due to its low frequency and atypical presentation, GP constitutes a true diagnostic and therapeutic challenge. Appropriate clinicoradiological and histopathological correlation is essential for accurate diagnosis and management [10,11,14]. The aim of this report is to present two cases of GP, highlighting their clinical, radiological, and histopathological features, diagnostic challenges, and surgical management. These cases provide an educational perspective on this uncommon variant and emphasize the importance of considering GP in the differential diagnosis of large, long-standing superficial masses.

## Case presentation

Case report 1

A 22-year-old woman with no significant past medical history presented with a right suprascapular mass of chronic evolution. Physical examination revealed a pear-shaped neoformation measuring 14 x 8 x 5 cm, skin-colored, with well-defined borders, rock-hard consistency, mobility on palpation, and absence of tenderness. The lesion had shown slow and progressive growth over a three-year period (Figure 1).

**Figure 1:**
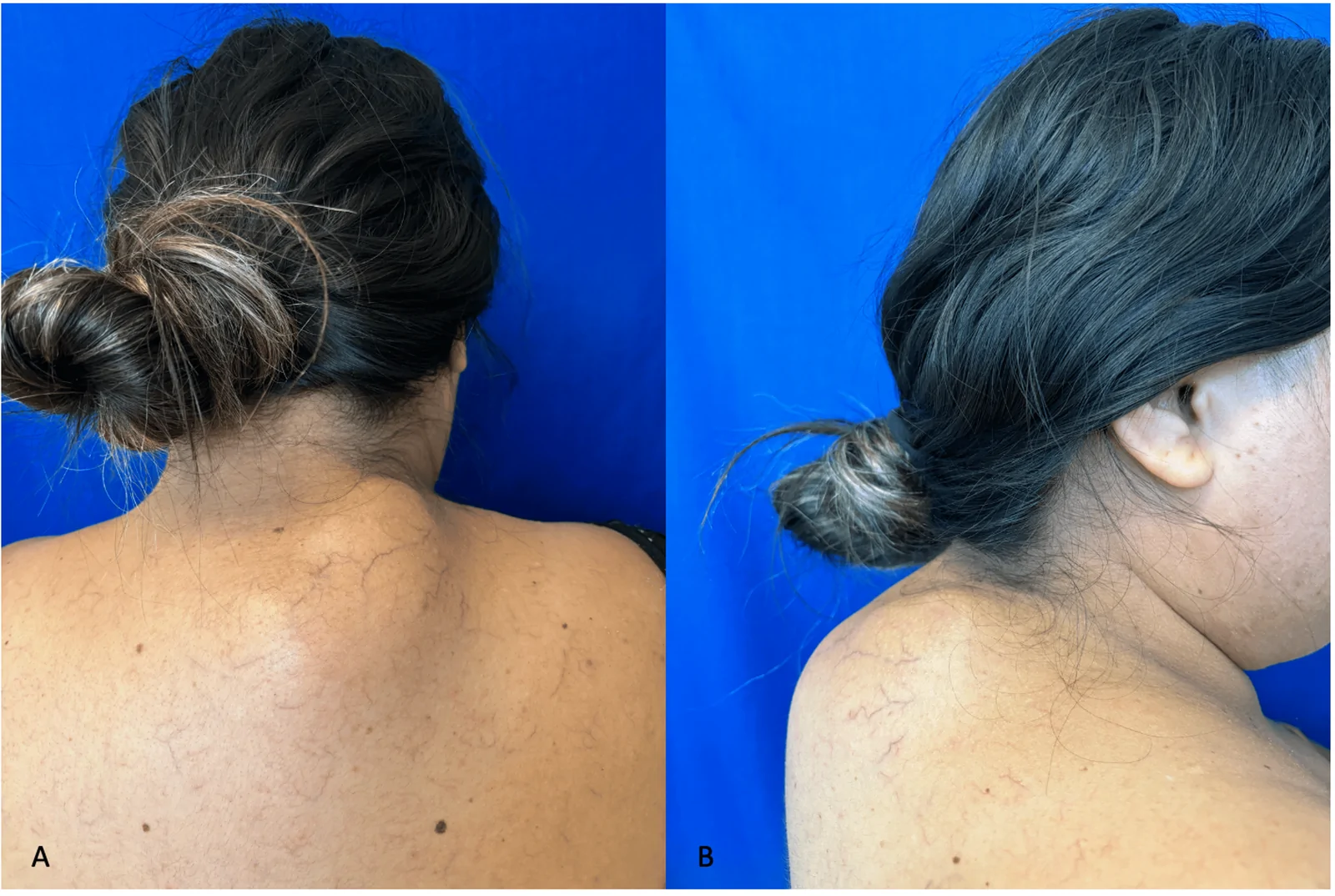
(A) Posterior view and (B) right lateral view of the suprascapular region, showing a subcutaneous neoplasm measuring approximately 14 × 8 × 5 cm, with well-defined borders and superficial telangiectasias, without ulceration.

Magnetic resonance imaging demonstrated a well-circumscribed lesion with iso- to hypointense signal intensity and foci suggestive of calcification. Based on the clinical and radiological findings, a long-standing neoplasm was suspected, with giant calcifying epithelioma considered the most likely diagnosis. Complete surgical excision was performed through an S-shaped incision, with complete excision of the tumor capsule, followed by layered closure.

Histopathological examination with hematoxylin and eosin staining revealed extensive basophilic areas corresponding to calcium deposits, abundant anucleated ghost cells, and scarce basaloid cells, with tumor-free surgical margins. These findings confirmed the diagnosis of calcifying epithelioma in an advanced stage of evolution (Figure 2).

**Figure 2:**
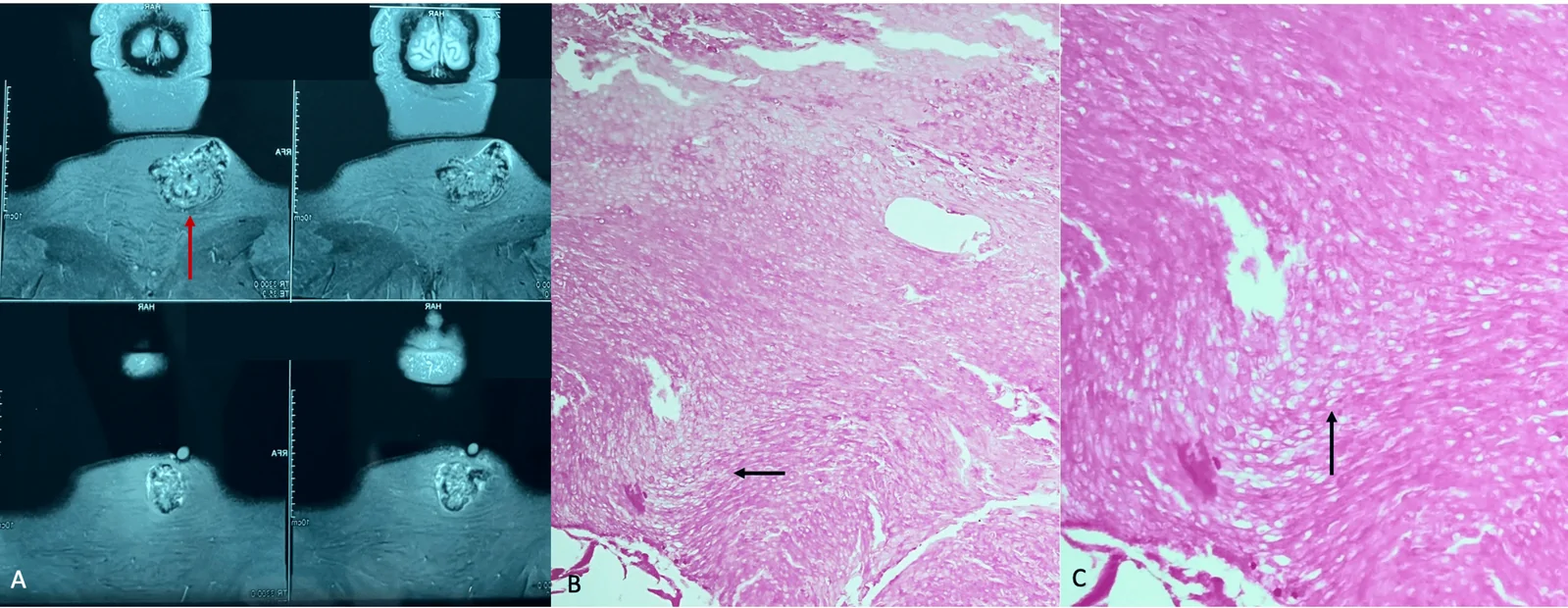
(A) Magnetic resonance imaging (coronal and axial views) demonstrating a well-circumscribed subcutaneous lesion in the suprascapular region, with iso- to hypointense signal intensity and focal hypointense areas consistent with calcification, without infiltration of the underlying muscular planes (red arrow). (B) Hematoxylin and eosin (H&E)-stained histopathological section showing a lesion composed of anucleate ghost cells with extensive areas of keratinization and calcification (black arrow). (C) Higher-magnification view revealing abundant ghost cells with loss of nuclear detail (black arrow), arranged in compact sheets, without significant cytologic atypia or mitotic activity, findings consistent with a mature or regressive pilomatrixoma.

The postoperative course was favorable, without immediate complications and adequate wound healing. After eight months of follow-up, no evidence of recurrence was observed (Figures 3, 4).

**Figure 3:**
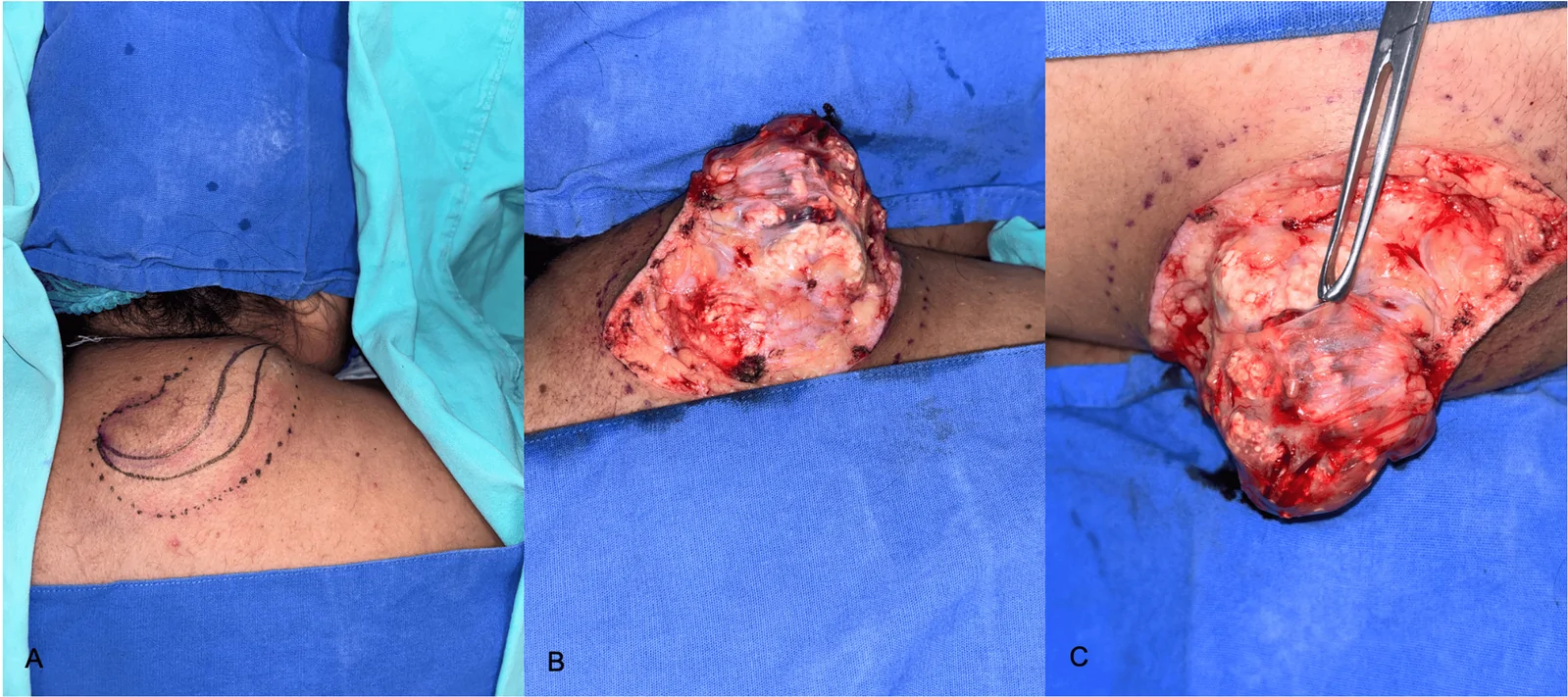
(A) Preoperative marking of the lesion in the suprascapular region, outlining the surgical margins. (B) Intraoperative view following lesion dissection. (C) Exposure of the surgical specimen during resection, demonstrating a partially fibrous capsule and relatively well-defined cleavage planes, without apparent infiltration of deeper structures.

**Figure 4:**
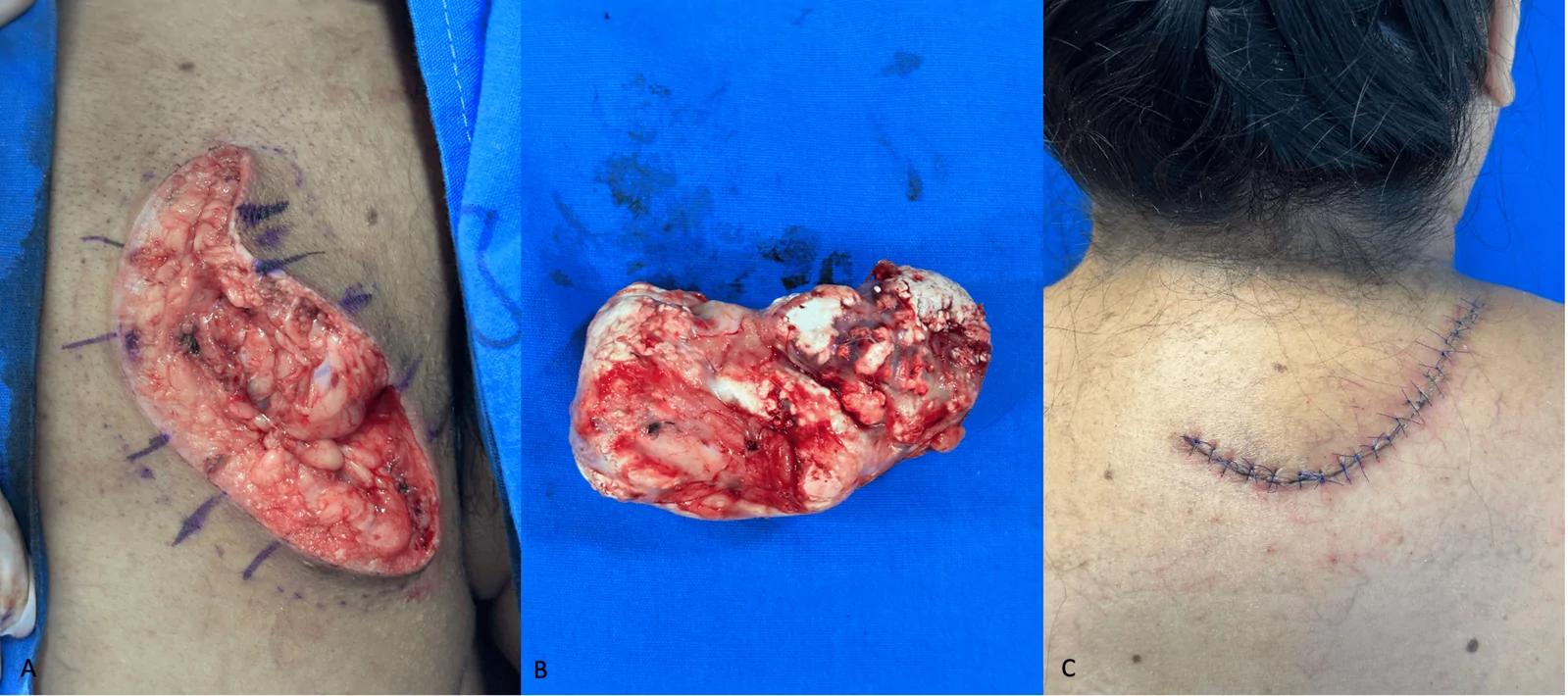
(A) Surgical bed following complete excision, demonstrating the postoperative defect. (B) Gross surgical specimen measuring approximately 16 cm in its greatest dimension, with a lobulated appearance, irregular surface, whitish-yellow areas, and focal calcifications. (C) Immediate postoperative appearance after primary wound closure.

Case report 2

A 54-year-old male with no relevant medical history presented with a left retroauricular mass measuring 6.5 × 4.5 × 3 cm, characterized by a skin-colored surface, well-defined borders, and a stony-hard consistency, with a four-year history of slow, progressive growth. Based on the clinical findings, pilomatrixoma was considered the main diagnostic impression.

Complete surgical excision of the lesion with layered closure was performed. Histopathological examination confirmed the diagnosis of pilomatrixoma, with tumor-free surgical margins, confirming complete excision of the lesion. The postoperative course was uneventful, with satisfactory wound healing and no immediate complications. At one-month postoperative follow-up, no evidence of recurrence was observed (Figure 5).

**Figure 5:**
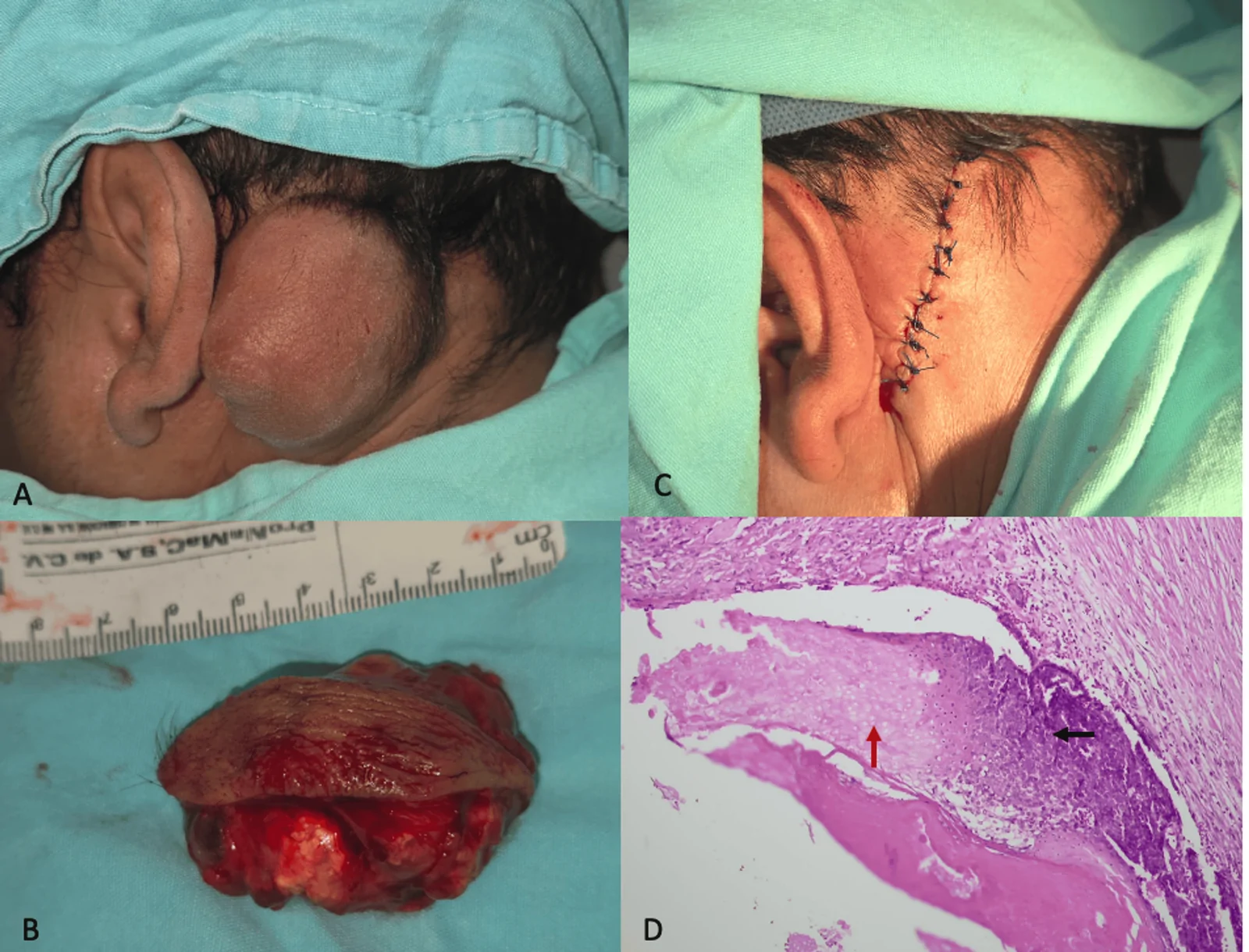
(A) Preoperative right lateral view of the retroauricular region showing a well-circumscribed, dome-shaped subcutaneous mass with smooth overlying skin and no evidence of ulceration. (B) Gross surgical specimen after excision, demonstrating a lobulated tumor with an irregular external surface and focal areas of calcification. (C) Immediate postoperative appearance following complete excision and primary closure, showing a linear retroauricular incision with satisfactory wound approximation. (D) Hematoxylin and eosin-stained section showing foci of transition from basaloid cells (black arrow) to anucleate shadow (ghost) cells (red arrow), accompanied by focal inflammatory infiltrate. The lesion is surrounded by a fibrous capsule, consistent with the characteristic histopathological features of pilomatrixoma. (H&E stain, original magnification ×40.)

## Discussion

The present cases highlight the clinicopathological variability of pilomatrixoma with respect to size, location, age at presentation, and degree of histopathological maturation. While one lesion presented as a large suprascapular tumor in a young woman, the other manifested as a retroauricular lesion of prolonged evolution in an older male patient. These findings underscore the broad clinical spectrum of this entity and its potential to present with features that may complicate clinical diagnosis.  

GP is an uncommon clinical variant of pilomatrixoma, accounting for approximately 10% of reported cases [8]. The dimensional criteria for defining GP remains controversial. While some authors consider lesions larger than 3 cm, most currently accept a cutoff value of >5 cm [8,16]. From a clinical perspective, its behaviour differs from that of classic pilomatrixoma. GP has been more frequently described in non-cephalic regions, particularly the trunk, the shoulders, and infraclavicular areas, in contrast to the head and neck predilection observed in conventional pilomatrixoma [9,10,13]. Nevertheless, in the literature it has been observed that the clinical presentation can be presented in any topography (Table 1). Clinically, it presents as a firm to rock-hard mass with slow progressive growth, which in advanced stages may develop superficial changes such as telangiectasias, cutaneous irregularities, or, in some cases, ulceration [9,11].

**Table 1:** Largest Giant Pilomatrixomas Reported in the Literature (2012–2026)

Author / Year	Age (years) / Sex (Male/Female)	Location	Size
Nardeshad et al. 2012 [17]	28 / Male	Cheek	5 x 3 cm
Egger et al., 2020 [18]	12 / Male	Posterior shoulder	6 × 4.5 cm
Wydadi et al., 2021 [19]	69 / Male	Scalp	17 × 9 cm
Duarte et al., 2024 [20]	75 / Female	Scalp	21 x 16.5 x 16 cm
Gongidi et al., 2015 [8]	52 / Male	Posterior chest	24 x 21 x 9 cm
Beattie et al., 2018 [21]	17 / Male	Upper back	34 × 21 × 17 cm

Regarding epidemiological characteristics, a slight male predominance and higher frequency among older adults, particularly during the sixth and seventh decades of life, have been reported [22-24]. Nevertheless, pilomatrixoma may occur across a broad age spectrum. Our cases illustrate this demographic diversity, involving a young female patient with large suprascapular lesion and an older male patient presenting with a long-standing retroauricular tumor. The differential diagnosis of GP is broad and includes both benign and malignant lesions. Benign lesions such as epidermoid cysts and calcified lipomas may clinically resemble GP, particularly when presenting as large, firm, slow-growing masses. Among malignant neoplasms, dermatofibrosarcoma protuberans and pilomatrical carcinoma should be considered, particularly in lesions with rapid growth, ulceration, or other atypical clinical features [9,14,18,22]. Proliferating trichilemmal tumors and other calcified soft-tissue tumors may also be included in the differential diagnosis because of their potential for large size, keratinization, and/or prominent calcification. Although these entities may share overlapping clinical and imaging features with GP, histopathological examination is essential for definitive diagnosis and differentiation. In particular, pilomatrical carcinoma is distinguished from GP by the presence of malignant features such as cytologic atypia, infiltrative growth, and increased mitotic activity, whereas the characteristic basaloid cells, ghost cells, and calcification support the diagnosis of GP. This broad spectrum of potential diagnoses may partially explain the high rate of initially incorrect diagnoses reported in the literature.

Definitive diagnosis is established histopathologically. Classically, pilomatrixoma exhibits a biphasic architecture characterized by peripheral basaloid cells, transitional cells, and central areas composed of anucleated ghost cells. However, in long-standing lesions, such as the case presented herein, a marked reduction or even absence of basaloid cells is frequently observed, with predominance of ghost cells and extensive calcium deposits, corresponding to late stages of tumor maturation [12,15]. Additionally, secondary ossification and foreign body-type inflammatory reactions associated with the release of keratinous material may also be identified [12]. Imaging studies represent an important complementary diagnostic tool. Computed tomography and magnetic resonance imaging usually demonstrate well-circumscribed lesions with central calcification foci, poor vascularization, and limited extension into deeper planes, findings that support their benign nature [9]. In this regard, their utility lies not only in guiding the diagnosis but also in facilitating appropriate surgical planning by accurately delineating lesion extent and its relationship with adjacent structures, thereby favoring complete excision. Furthermore, chronic inflammatory response and reaction to released keratin have been proposed as contributing factors for the progressive enlargement of these lesions, potentially explaining the development of giant variants [11].

Complete surgical excision with free margins remains the treatment of choice. The recurrence rate is low, estimated at approximately 0.3%, although it increases significantly in cases of incomplete resection [23]. Importantly, although uncommon, recurrences have occasionally been associated with transformation into pilomatricial carcinoma, a malignant neoplasm characterized by infiltrative growth, cytological atypia, high mitotic activity, and metastatic potential [3,24]. Therefore, complete excision and long-term clinical follow-up are essential for early detection of changes in the biological behavior of the lesion.

Finally, analysis of the cases reported in the literature, summarized in Table 1, provides further context regarding the magnitude of this variant. The table includes some of the largest giant pilomatrixomas documented, with dimensions ranging from 5 cm to more than 30 cm in greatest diameter. A predominance among male patients and location on the trunk and scalp is evident. 

## Conclusions

The presented cases demonstrate the clinical variability of GP and the diagnostic challenges posed by its large size and long-standing evolution, features that may lead to confusion with other soft tissue neoplasms. In both cases, correlation of the clinical assessment with imaging studies and histopathological examination allowed an accurate diagnosis and successful surgical management, with no evidence of recurrence during follow-up. These cases also highlight the importance of considering GP in the differential diagnosis of slow-growing, long-standing superficial masses with a stony-hard consistency, as its early recognition may facilitate appropriate surgical planning and contribute to timely and appropriate management of this uncommon variant. Therefore, clinicians should maintain a high index of suspicion for GP when evaluating large, long-standing, calcified superficial masses, and histopathological examination remains essential for definitive diagnosis and exclusion of malignant mimickers. Complete surgical excision remains the preferred therapeutic approach and allows definitive histopathological diagnosis. Although based on two cases, this report adds to the limited literature on GP and provides further clinical and histopathological insight into this rare variant.
